# New antiretrovirals

**DOI:** 10.1186/1742-4690-9-S1-I7

**Published:** 2012-05-25

**Authors:** Roy Gulick

**Affiliations:** 1 Weill Medical College of Infectious Diseases, New York, USA

## 

There are 26 approved antiretroviral drugs available in 2012 in 6 mechanistic classes: reverse transcripatase inhibitors (both nucleosides and non-nucleosides), protease inhibitors, entry inhibitors (both fusion inhibitors and CCR5 receptor antagonists), and integrase inhibitors. Current antiretroviral therapy combinations dramatically decrease HIV-related morbidity and mortality. However, despite these advances, some current antiretroviral regimens may be inconvenient, toxic, and/or have suboptimal antiretroviral activity, particularly against drug-resistant viruses. Thus, newer compounds are needed that improve convenience and tolerability, reduce toxicity, and improve antiretroviral activity, particularly against drug-resistant viruses. Additionally, new drugs may better penetrate tissue reservoirs (e.g. genital tract, central nervous system), exploit new targets with new mechanisms of action, or be administered in new formulations.

There are a number of HIV investigational drugs in development currently. These include a new pharmacokinetic “boosting” agent, cobicistat (GS-9350) and newer antiretroviral agents in a number of classes, including new nucleoside reverse transcriptase inhibitors, non-nucleoside reverse transcriptase inhibitors, protease inhibitors, entry inhibitors, and integrase inhibitors. Of those in the pipeline, a few compounds are in advanced stages of development: the nucleoside analogue GS-7340, a pro-drug of tenofovir (phase 2); and the integrase inhibitors, elvitegravir (phase 3 completed) and dolutegravir (phase 3). In addition, there are drugs with new mechanisms of action in development, including the CD4 attachment inhibitor, BMS-663068 (phase 2), and the CCR5 antagonist, cenicriviroc (phase 2).

Probably the greatest need in the HIV clinic today is compounds that have activity against multidrug-resistant viral strains. Another important need is alternative one-pill, once-daily formulations for both initial and subsequent regimens. However, the clinical use of these newer agents will depend on the results of phase 3 clinical trials, and the timeline for development and availability.

